# Exploring Racial Disparities in Awareness and Perceptions of Oncology Clinical Trials: Cross-Sectional Analysis of Baseline Data From the mychoice Study

**DOI:** 10.2196/56048

**Published:** 2024-09-30

**Authors:** Ariel Hoadley, Linda Fleisher, Cassidy Kenny, Patrick JA Kelly, Xinrui Ma, Jingwei Wu, Carmen Guerra, Amy E Leader, Mohammed Alhajji, Paul D’Avanzo, Zoe Landau, Sarah Bauerle Bass

**Affiliations:** 1 Department of Social and Behavioral Sciences Temple University College of Public Health Philadelphia, PA United States; 2 Cancer Prevention and Control Fox Chase Cancer Center Philadelphia, PA United States; 3 Department of Epidemiology and Biostatistics Temple University College of Public Health Philadelphia, PA United States; 4 Abramson Cancer Center University of Pennsylvania Medical Center Philadelphia, PA United States; 5 Division of Population Science, Department of Medical Oncology Sidney Kimmel Cancer Center Thomas Jefferson University Philadelphia, PA United States

**Keywords:** oncology clinical trial, cancer, decision-making, racial disparity, medical mistrust

## Abstract

**Background:**

Black/African American adults are underrepresented in oncology clinical trials in the United States, despite efforts at narrowing this disparity.

**Objective:**

This study aims to explore differences in how Black/African American oncology patients perceive clinical trials to improve support for the clinical trial participation decision-making process.

**Methods:**

As part of a larger randomized controlled trial, a total of 244 adult oncology patients receiving active treatment or follow-up care completed a cross-sectional baseline survey on sociodemographic characteristics, clinical trial knowledge, health literacy, perceptions of cancer clinical trials, patient activation, patient advocacy, health care self-efficacy, decisional conflict, and clinical trial intentions. Self-reported race was dichotomized into Black/African American and non–Black/African American. As appropriate, 2-tailed *t* tests and chi-square tests of independence were used to examine differences between groups.

**Results:**

Black/African American participants had lower clinical trial knowledge (*P*=.006), lower health literacy (*P*<.001), and more medical mistrust (all *P* values <.05) than non–Black/African American participants. While intentions to participate in a clinical trial, if offered, did not vary between Black/African American and non–Black/African American participants, Black/African American participants indicated lower awareness of clinical trials, fewer benefits of clinical trials, and more uncertainty around clinical trial decision-making (all *P* values <.05). There were no differences for other variables.

**Conclusions:**

Despite no significant differences in intent to participate in a clinical trial if offered and high overall trust in individual health care providers among both groups, beliefs persist about barriers to and benefits of clinical trial participation among Black/African American patients. Findings highlight specific ways that education and resources about clinical trials could be tailored to better suit the informational and decision-making needs and preferences of Black/African American oncology patients.

## Introduction

### Background

The underrepresentation of racial and ethnic minoritized populations in cancer clinical trials is well-established [1-4], particularly among Black/African American adults [5-10]. Despite federal initiatives and policies aimed at increasing cancer clinical trial enrollment and participation rates of underrepresented groups, rates have not improved among people from racial and ethnic minoritized groups, and in some cases, the rates have even declined [11]. Attributable to factors across multiple levels of influence [12], the underrepresentation of Black/African American adults in cancer clinical trials means that drugs and interventions are developed, tested, and disseminated to populations not reflective of the broader US cancer population, perpetuating health inequities [13].

For example, 1 study found that Black/African American adults comprised only 7.4% of all participants in US Food and Drug Administration clinical trials that led to new, approved cancer drugs from 2014 to 2018 [8]. The participation-to-prevalence ratio reflects the representation of Black/African American adults in the clinical trial population relative to the general cancer population, where a ratio of 1 means there is identical or equal representation between groups. Across cancer types, the estimated participation-to-prevalence ratio for Black/African American US adults was 0.31, indicating significant underrepresentation in clinical trials that result in Food and Drug Administration approvals for cancer drugs [8]. Importantly, Black/African American adults are also less likely to participate in trials of novel treatments and technologies, such as precision oncology [4,14]. These disproportionately low rates of clinical trial participation among racial and ethnic minorities result in limited understanding by medical professionals and the greater research community of how well new diagnostic technology, treatment options, and supportive care services are working for racial and ethnic minorities in comparison to the predominantly White clinical trial participant population [15,16].

In addition to underrepresentation in cancer clinical trials, inequities in cancer care and survival rates persist [17-19]. Greater inclusion of Black/African American patients in cancer clinical trials is, therefore, essential to design and test interventions to address inequities in cancer care among Black/African American patients. For example, non-Hispanic Black/African American patients have significantly greater cancer diagnosis delay [17], treatment delay [17], and likelihood of diagnosis at an advanced cancer stage [18] compared with non-Hispanic White patients. Even after accounting for cancer stage, cancer type, and other relevant covariates, Black/African American patients still have significantly lower survival rates than White patients [19].

Prior studies have found that non-Hispanic, Black/African American patients have less awareness of cancer clinical trials and hold specific attitudes and beliefs about trial participation relative to non-Hispanic, White patients [20,21]. For example, in a qualitative study of Black/African American cancer survivors who received cancer treatment at a safety-net hospital, the primary clinical trial participation barriers were (1) limited knowledge and understanding of cancer clinical trials and (2) medical mistrust, fears, and other negative perceptions of cancer clinical trials. Participants also described wanting a peer (cancer survivor of a concordant race or ethnicity group) patient navigator who was well-versed in clinical trials knowledge and who could provide other forms of social support (eg, social or emotional, faith-based or spiritual, and instrumental support) [22]. These results were consistent with other studies emphasizing the roles of knowledge or awareness, medical mistrust, and social support in clinical trial enrollment; study participation; and retention over time [23-25].

Other specific attitudes held by Black/African American patients with cancer more than White patients include lower perceived cancer susceptibility and greater doubt about the usefulness and feasibility of translating cancer clinical trial results into clinical practice [23]. Other patient-level factors associated with less knowledge and awareness of cancer clinical trials include living in a rural area [26], living farther away from universities or large hospital networks [27], older age [28], limited English language proficiency [29], lower educational attainment [21], and less annual household income [21]. Conversely, greater cancer clinical trial knowledge and the likelihood of trial participation are associated with a prior cancer diagnosis [30], having a routine source of health care (ie, primary care access) [31], and higher educational attainment [30]. Trial populations’ clinical knowledge and awareness are essential constructs for researchers to be aware of because the quality of communication between clinical trial staff and prospective trial participants is, in part, dependent upon patients’ clinical trial knowledge and confidence [32].

Negative attitudes toward cancer clinical trials, particularly having greater concerns, are associated with cancer fatalism [33]. Other concerns cited by Black/African American patients with cancer associated with decreased cancer clinical trial intentions are greater fear of the unknown [33], fear of death [33], prior negative health care or clinical trial experiences [22,34,35], fear of receiving an inferior treatment or placebo [22], lower health literacy [36,37], anticipated discrimination [33], and medical mistrust [33,38]. Structural racism, historical injustices, and unethical research practices have disproportionately affected Black/African American people and have perpetuated concerns of anticipated mistreatment by research personnel and broader medical mistrust [39-41]. However, levels of cancer-related knowledge and specific attitudes toward cancer clinical trials are associated with cancer clinical trial participation rates among Black/African American patients with cancer. For example, a qualitative study among Black men found that perceptions of greater research integrity and transparency were positively associated with willingness to participate in prostate cancer surveillance screening and clinical trials [38]. Other factors positively associated with willingness to participate in cancer research were having a family history of cancer, seeing greater value in screening and cancer prevention, and having more interest in learning about cancer and other health-related information [38].

At the interpersonal level, Black/African American patients with cancer have differential access to cancer clinical trial information attributable to provider biases and patient-provider communication quality. For example, clinical trials are often initially discussed with patients by their health care providers, but provider bias, including racism and discrimination, results in less information sharing and discussion about cancer screenings, clinical trials, and cancer treatment options for Black/African American patients than for White patients [42]. At the clinic level, limited hiring of providers with language fluency beyond English reduces clinic access and decreases the feasibility of within-session information sharing about clinical trials for patients and families with limited English language proficiency [43]. Importantly, many Black/African American patients report not being offered a trial during their cancer care [44-46], despite overall positive perceptions of clinical trials, further exacerbating the inequity [47].

Finally, it should be noted that individual-level awareness of clinical trials is only minimally helpful as an interventional target when structural and systemic factors more strongly drive participation rates. For example, studies have repeatedly demonstrated that some of the greatest barriers to clinical trial enrollment are inequitable clinical trial referrals and enrollment practices [48] and stringent trial eligibility criteria [49-52]. Recent programs and initiatives implemented to increase awareness of cancer clinical trials among Black/African American patients have recognized that awareness must be addressed at multiple levels of influence to advance health equity. For example, a June 2022 article published by the American Society of Clinical Oncology suggests that clinics and health care facilities use 1 of 2 standardized clinic self-assessment tools to review their enrollment practices and patient-, provider-, and system-level barriers to clinical trial enrollment [52-56].

This study is a cross-sectional analysis of the baseline data from a parent randomized controlled trial (RCT) designed to evaluate the impact of a multicultural, clinical trial preparatory digital health tool (mychoice) or standard National Cancer Institute information for patients with cancer. mychoice was conceptualized and developed by a team of investigators at Fox Chase Cancer Center and the Temple University College of Public Health through extensive formative research with Black/African American patients, expertise in health disparities and clinical trial participation, commercial marketing techniques (perceptual mapping and vector message modeling), and best practices in digital health and patient engagement [47,57]. Although founded on clinical trial participation barriers significant to underrepresented patients, the tool is designed to be appropriate for all patients with cancer and to represent diverse patient perspectives.

### Objectives

A diverse sample of patients enrolled in the parent RCT completed a baseline survey before viewing the decision-making tool, providing an opportunity to explore racial disparities in a variety of factors previously linked to clinical trial participation rates and the clinical trial participation decision-making process. On the basis of the conducted formative work with Black/African American patients to inform the digital health tool used in the parent RCT, this study sought to confirm whether factors identified in the formative work were, in fact, salient to Black/African American patients with cancer relative to non–Black/African American patients with cancer at baseline. Findings will help explain Black/African American versus non–Black/African American participant responses to the culturally tailored, clinical trial decision-making tool and also help identify factors that could help further refine the decision-making tool. In addition, findings can be used to tailor and prioritize topics in provider education and training to better support the needs of Black/African American patients with cancer in cancer clinical trial decision-making.

## Methods

### Participants

The analytical sample at baseline included patients with cancer from 4 leading cancer centers in Philadelphia (Fox Chase Cancer Center, Temple University Hospital, University of Pennsylvania’s Abramson Cancer Center, and Thomas Jefferson University’s Sidney Kimmel Cancer Center) who consented to participate in the parent RCT (NCT03427177) and completed the baseline survey. Moreover, 3 of the 4 recruitment sites are National Cancer Institute–designated cancer centers. Eligible patients were actively being treated for cancer or in follow-up care (ie, within 6 months of definitive treatment), aged ≥18 years, able to speak and read English, and had not participated in a therapeutic clinical trial. The parent RCT had been planned to enroll 270 participants. In total, 257 participants consented and 249 (96.9%) completed the baseline survey. Patients of all racial and ethnic groups were eligible for the RCT, but only 244 (98%) of the 249 completed baselines reported valid or nonmissing data for their race and were analyzed in this study.

### Instruments

#### Overview

The survey was developed using both validated instruments and study-related measures from formative work, including both qualitative interviews and surveys with Black/African American patients with cancer [47,57-59]. Variables included in the present analyses were sociodemographic characteristics (ie, age, race, ethnicity, gender, income, educational attainment, insurance type, and cohabitation status), dichotomized race group (Black/African American vs non–Black/African American), clinical characteristics (ie, cancer stage and treatment status), general clinical trial knowledge, health literacy, cancer clinical trial perceptions (awareness, benefits, concerns, and cancer and health care experiences beliefs about health care providers and health), patient activation in cancer care, patient self-advocacy, self-efficacy in health care interactions, decisional conflict, and clinical trial intentions.

#### General Knowledge of Clinical Trials

General knowledge of clinical trials was assessed using 16 revised items from Knowledge of Clinical Trials scale by Campbell et al [60]. Response options were “true” or “false” and were scored for accuracy. Scores were generated using the percentage of questions answered correctly, ranging from 0% to 100%.

#### Health Literacy

Health literacy was assessed with a single item from the Single Item Literacy Screener, which specifically identifies adults who may need assistance reading and understanding health materials [61]. The item says, “How often do you need to have someone help you when you read instructions, pamphlets, or other written material from your doctor or pharmacy?” Response options were rated on a 5-point Likert scale, ranging from a score of “1” reflecting “never” to “5” reflecting “always.” On the basis of psychometric testing, scores >“2” reflect people with limited health literacy in reading and comprehending written health information [61].

#### Cancer Clinical Trial Perceptions

Perceptions of cancer clinical trials were evaluated using 48 items developed by the primary investigators through formative work, reflecting domains of (1) awareness, (2) benefits, (3) concerns, (4) cancer and health care experiences, and (5) beliefs about health care providers and health [47,57-59]. Response options were rated on an 11-point Likert scale ranging from 0 to 10 where “0” indicated strong disagreement and “10” indicated strong agreement. Item-level analyses were conducted in this study.

#### Patient Activation in Cancer Care

Patient activation for cancer care decision-making was measured with 10-item Decisional Engagement Scale [62]. This instrument was developed specifically to understand patients’ level of involvement in their cancer care and engagement with active decision-making processes around treatment and care options [62]. Response options were rated on an 11-point Likert scale, ranging from 0 to 10 where “0” meant “doesn’t describe you at all” and “10” meant “perfectly describes you.” In psychometric evaluation, the 10-item Decisional Engagement Scale has demonstrated strong factor structure, reliability, and concurrent validity with health-related quality of life, shared decision-making preferences, and clarity about cancer care preferences [62].

#### Patient Self-Advocacy

Patient self-advocacy was measured with 12-item Patient Self-Advocacy Scale [63]. Response options were rated on an 11-point Likert scale, ranging from 0 to 10. In addition, 1 item (“I don’t get what I need from my physician because I am not assertive enough”) was reverse coded before calculating an average summary score. The scale has demonstrated good internal consistency, construct validity, and criterion validity [63].

#### Health Care Self-Efficacy

Self-efficacy to engage with health care providers was measured with 10-item Perceived Self-Efficacy in Patient-Physical Interactions scale [64]. Items asked about confidence to do specific health care–related tasks, such as confidence to get a physician to listen to them, confidence in ability to know what questions to ask a physician, and confidence in ability to get a physician to take their health concerns seriously. Response options ranged from 1 to 5, where “1” indicated least confidence and “5” indicated most confidence [64].

#### Decisional Conflict

Decisional conflict about clinical trial participation was measured with 13-item Decisional Conflict scale proposed by O’Connor [65]. Response options were rated on a 5-point Likert scale, ranging from 0 to 4 where “0” reflected “strongly agree” and “4” reflected “strongly disagree.” Scoring of 4 subscales (uncertainty, informed, value clarity, and decision support) was done by summing the items within the subscale, dividing by the number of items within that subscale, and multiplying by 25. This resulted in a score ranging from 0 to 100. A total score for all items was also calculated by summing all items, dividing by 13, and multiplying by 25. This, too, led to a total score ranging from 0 to 100. In psychometric testing, the scale had good discriminant validity between those who choose versus those who do not choose to engage in a health behavior. Other psychometric properties were determined to be acceptable [65].

#### Clinical Trial Participation Intentions

Intentions to participate in a cancer clinical trial were assessed with a single, modified item from the Choice Predisposition Scale proposed by O’Connor [66]. The item read, “We would like to know what your opinion is about your cancer treatment options at present. When your doctor asks you to make a choice about treatment methods, please indicate how strongly you agree or disagree that you would choose to participate in a clinical trial, if offered.” Response options ranged from 0 to 10, where “0” indicated strongly disagree,” a “5” meant “neither agree nor disagree,” and “10” indicated “strongly agree.” This scale has good psychometric properties, such as high test-retest validity, good construct validity, high sensitivity to change, and discriminant validity [66].

### Procedures

Prospective participants were screened for eligibility (aged ≥18 years, cancer diagnosis, receiving current or follow-up care, English speaking, and had not previously participated in a clinical trial). Participants provided verbal informed consent either in person or over the phone. Consent was verified via an e-consent using REDCap (Research Electronic Data Capture; Vanderbilt University), a web-based application developed to capture data for research [67,68]. Consented patients were randomized to intervention conditions via REDCap and completed a baseline survey prior to viewing any intervention content. The baseline survey assessments were web-based and were conducted through REDCap. Patients could either complete the study at the hospital using a study iPad (Apple Inc) or at home on their own devices. The baseline survey took approximately 45 minutes.

### Statistical Analysis

Univariate statistics using means, SDs, and percentages are presented to characterize the participant sample. Differences in sociodemographic and clinical characteristics between dichotomous race groups (ie, Black/African American and non–Black/African American patients) were evaluated using chi-square tests of independence and independent sample 2-tailed *t* tests, as appropriate. Independent sample *t* tests were also used to examine for differences between Black/African American and non–Black/African American patients’ clinical trial knowledge, attitudes toward cancer clinical trials, and intentions to participate in a clinical trial. While some variables (eg, health literacy and self-efficacy in health care interactions) were highly skewed, *t* tests were still used as opposed to nonparametric testing because *t* tests are robust to skewed distributions when the sample size is >200 [69]. Homogeneity of variances between groups was evaluated for each item before running independent samples *t* tests, and the appropriate *t* test assumptions were applied accordingly. All data analyses were conducted in StataSE (version 17.0; StataCorp).

### Ethical Considerations

The study protocol was approved by the Fox Chase Cancer Center’s institutional review board (#17-8013). All procedures involving human participants were in accordance with the ethical standards of the institutional or national research committee and with the 1964 Declaration of Helsinki and its later amendments or comparable ethical standards. All participants provided verbal informed consent. Verification of consent with e-consent and all other study data were collected in REDCap, a secure web-based application developed to collect and store research data [67,68]. To protect participants’ privacy, the data were coded before analysis using unique participant study identifiers and no direct identifiers were in the analytic data set. Participants were compensated US $25 for completing the baseline survey, educational intervention, and the posttest survey. However, this paper describes results from the baseline survey data only.

## Results

### Overview

Table 1 compares sociodemographic and clinical characteristics by dichotomous race group. Tables 2-4 show results of all remaining independent sample *t* tests for differences in average general clinical trials knowledge, health literacy, perceptions of cancer clinical trials, patient activation, patient advocacy, health care self-efficacy, decisional conflict, and clinical trial intentions by race group.

**Table 1:** Sociodemographic and clinical characteristics of study participants by race at baseline (N=244).

Characteristics			Total^a^ (N=244)		Non–Black/African American (n=149)		Black/African American (n=95)		Chi-square (*df*) or *t* test (*df*)			*P* value	
**Gender, n (%)**										7.465 (1)			.006
	Female	154 (63.1)		84 (56.4)		70 (73.7)							
	Male	90 (36.9)		65 (43.6)		25 (26.3)							
**Age (y), mean (SD)**			60.89 (10.24)		61.62 (11.13)		59.28 (10.24)		1.866 (242)			.06	
**Educational attainment, n (%)**										59.509 (2)			<.001
	Less than high school	29 (11.9)		8 (5.4)		21 (22.1)							
	High school or GED^b^	74 (30.3)		26 (17.4)		48 (50.5)							
	Some college or more	141 (57.8)		115 (77.2)		26 (27.4)							
**Insurance type, n (%)**										17.379 (1)			<.001
	Private	92 (38.3)		72 (48.7)		20 (21.7)							
	Medicare or Medicaid	148 (61.7)		76 (51.3)		72 (78.3)							
**Race, n (%)**										—^c^			—
	American Indian or Alaskan Native	1 (0.4)		1 (0.7)		0 (0)							
	Asian	2 (0.8)		2 (1.3)		0 (0)							
	Black/African American	95 (38.9)		0 (0)		95 (100)							
	White	136 (55.7)		136 (91.3)		0 (0)							
	More than 1 race	10 (4.1)		10 (6.7)		0 (0)							
**Ethnicity, n (%)**										2.775 (1)			.09^d^
	Hispanic/Latino	10 (4.7)		9 (6.5)		1 (1.4)							
	Non–Hispanic/Latino	202 (95.3)		130 (93.5)		72 (98.6)							
**Annual household income (US $), n (%)**										78.660 (2)			<.001
	<15,000	60 (26.7)		13 (9.5)		47 (53.4)							
	15,000-50,000	66 (29.3)		34 (24.8)		32 (36.4)							
	>50,000	99 (44)		90 (65.7)		9 (10.2)							
**Cohabitation status, n (%)**										2.697 (1)			.10
	No	55 (22.9)		28 (19.3)		27 (28.4)							
	Yes (lives with >1 people)	185 (77.1)		117 (80.7)		68 (71.6)							
**Cancer stage, n (%)**										0.020 (1)			.89
	Early	108 (56)		65 (55.6)		43 (56.6)							
	Late	85 (44)		52 (44.4)		33 (43.4)							
**Treatment status^e^, n (%)**										8.993 (1)			.003
	Receiving treatment	165 (73)		88 (65.7)		77 (83.7)							
	Receiving follow-up care	61 (27)		46 (34.3)		15 (16.3)							

^a^Percentages are rounded and, therefore, may not add up to 100%. Missing or invalid data were excluded from this table for insurance type (n=4), ethnicity (n=32), income (n=19), cohabitation status (n=4), cancer stage (n=51), and treatment status (n=18).

^b^GED: General Educational Development.

^c^Not applicable. This was because the table is split by binary race, so examining race by race is nonsensical.

^d^Fisher exact test was used when one or more of the expected cell counts was <5.

^e^Receiving treatment includes treatment types, such as chemotherapy, radiation, surgery, and any other types of cancer treatment. Follow-up care includes posttreatment care within 6 months of the last receipt of treatment.

**Table 2:** Baseline knowledge, health literacy, awareness of clinical trials for Black/African American versus non–Black/African American oncology patients (N=244).

			Non–Black/African American patients (n=149)				Black/African American patients (n=95)					*t* test (*df*)			*P* value
			Mean (SD)		95% CI		Mean (SD)		95% CI						
**General clinical trial knowledge^a^**			80.70 (14.73)		78.32-83.09		75.59 (12.86)		72.97-78.21		2.775 (242)			.006	
**Health literacy^b,c^**			1.47 (0.72)		1.35-1.58		2.06 (1.11)		1.84-2.29		–4.650 (145.36)			<.001	
**Awareness of clinical trials^d^**															
	I had heard about clinical trials before I was diagnosed.	7.61 (3.33)		7.06-8.15		5.19 (3.96)		4.39-6.01		5.075 (238)			<.001		
	I know where to get information about clinical trials.	5.30 (3.59)		4.71-5.89		4.44 (3.70)		3.68-5.19		1.801 (237)			.07		
	I know someone who has been part of a clinical trial who I can talk to about whether I should participate or not.	3.42 (3.69)		2.81-4.02		2.78 (3.53)		2.05-3.50		1.335 (238)			.18		
	I understand what clinical trials are and how they work.	4.41 (3.68)		3.80-5.02		4.31 (3.83)		3.52-5.09		0.204 (236)			.84		
	I do not have enough information about clinical trials to make a decision.	5.93 (3.55)		5.35-6.51		4.55 (3.63)		3.80-5.29		2.920 (238)			.004		
	My doctor gave me enough information to make a decision about being part of a clinical trial.	3.68 (3.70)		3.06-4.29		3.76 (3.60)		3.02-4.51		–0.179 (233)			.86		
	Being part of a clinical trial means I get all or part of my medical care and medication for free^d^.	4.99 (3.38)		4.44-5.55		4.65 (4.15)		3.79-5.50		0.677 (167.75)			.50		

^a^Clinical trials knowledge was a percentage ranging from 0 to 100.

^b^Health literacy ranged from 0 to 4, where higher values reflected lower health literacy.

^c^Variances were not equal between groups, so an independent sample *t* test with unequal variances was used.

^d^Response options for awareness items ranged from 0 to 10, where 0 indicated strong disagreement and 10 indicated strong agreement.

**Table 3:** Baseline perceived benefits and concerns about cancer clinical trials for Black/African American versus non–Black/African American oncology patients (N=244).

			Non–Black/African American patients (n=149)					Black/African American patients (n=95)					*t* test (*df*)			*P* value
			Mean (SD)		95% CI		Mean (SD)			95% CI						
**Benefits of clinical trial participation^a^**																
	I have a better chance of living longer if I am part of a clinical trial^b^.	5.37 (2.79)		4.91-5.83		4.30 (3.74)			3.53-5.06		2.396 (158.44)			.02		
	Being part of a clinical trial improves my quality of life^b^.	5.16 (2.69)		4.72-5.60		4.24 (3.45)			3.53-4.95		2.184 (161.70)			.03		
	I believe the benefits of being in a clinical trial outweigh the possible side effects^b^.	5.37 (2.73)		4.92-5.82		4.43 (3.56)			3.71-5.16		2.176 (164.88)			.03		
	Being part of a clinical trial offers the best treatment available for my cancer^b^.	5.48 (2.87)		5.01-5.95		4.43 (3.78)			3.65-5.20		2.299 (160.87)			.02		
	Being part of a clinical trial can give a person a sense of purpose in life^b^.	6.15 (2.57)		5.73-6.57		4.76 (3.62)			4.01-5.50		3.246 (152.86)			.001		
	If my doctor said a clinical trial was the best option for me, I would follow their advice^b^.	7.91 (2.37)		7.52-8.30		6.93 (3.45)			6.22-7.63		2.429 (151.87)			.02		
	Being part of a clinical trial will improve my community’s trust in medical research^b^	5.94 (2.74)		5.49-6.39		5.17 (3.55)			4.44-5.90		1.785 (162.08)			.08		
	Being part of a clinical trial could help find a cure for cancer^b^.	8.24 (1.77)		7.95-8.53		6.99 (3.21)			6.34-7.64		3.468 (131.55)			<.001		
	Being part of a clinical trial would help my doctor and their research^b^.	8.05 (2.05)		7.71-8.38)		7.27 (3.18)			6.61-7.92		2.103 (141.08)			.04		
	Being part of a clinical trial could help my children or grandchildren in the future^b^.	8.27 (2.05)		7.94-8.61		7.27 (3.10)			6.63-7.91		2.760 (142.89)			.007		
	Being part of a clinical trial could help other people with my type of cancer^b^.	8.51 (1.82)		8.21-8.80		7.84 (2.73)			7.29-8.40		2.090 (148.21)			.04		
**Concerns of cancer clinical trial participation^a^**																
	I am worried that my health insurance won’t pay for me to be part of a clinical trial.	5.00 (3.31)		4.46-5.54		5.27 (3.66)			4.53-6.02		–0.602 (240)			.55		
	I believe that taking part in a clinical trial will cause more side effects than my current treatment.	4.47 (2.54)		4.06-4.89		3.82 (3.04)			3.20-4.44		1.798 (238)			.07		
	I believe that my medical care is not as good if I take part in a clinical trial.	2.77 (2.78)		2.31-3.23		2.70 (3.00)			2.09-3.32		0.181 (236)			.86		
	My religious beliefs could keep me from taking part in a clinical trial^b^.	0.58 (1.72)		0.30-0.86		1.73 (2.92)			1.13-2.33		–3.445 (132.69)			<.001		
	God has already decided what will happen so being part of a clinical trial would not help^b^.	0.84 (2.10)		0.50-1.19		2.95 (3.70)			2.19-3.71		–5.015 (132.15)			<.001		
	No one talked to me about being part of a clinical trial.	5.23 (4.07)		4.56-5.90		4.49 (3.80)			3.72-5.27		1.402 (236)			.16		
	I’m too upset about my cancer diagnosis to think about being part of a clinical trial.	1.70 (2.71)		1.25-2.14		2.23 (2.82)			1.66-2.81		–1.474 (237)			.14		
	I’m afraid I’ll get a sugar pill (placebo) instead of real medicine in a clinical trial.	4.00 (3.66)		3.40-4.60		2.72 (3.35)			2.03-3.40		2.750 (239)			.006		
	I’d worry that I’d be treated like a number, not a person, in a clinical trial.	2.66 (2.85)		2.20-3.13		2.84 (3.27)			2.18-3.51		–0.446 (239)			.66		
	I believe I would be treated like a “guinea pig” in a clinical trial^b^.	2.30 (2.74)		1.85-2.75		3.12 (3.39)			2.42-3.82		–1.944 (166.51)			.05		
	I believe I would not be told important information about my health if I was part of a clinical trial^b^.	2.23 (2.74)		1.79-2.69		2.67 (3.29)			2.00-3.34		–1.066 (172.53)			.29		

^a^Response options for perception items ranged from 0 to 10, where 0 indicated strong disagreement and 10 indicated strong agreement.

^b^Variances were not equal between groups, so independent sample *t* test with unequal variances was used.

**Table 4:** Baseline health care experiences, health care beliefs, patient self-advocacy, patient activation, health care self-efficacy, decisional conflict, and intentions to participate in cancer clinical trials for Black/African American versus non–Black/African American oncology patients (N=244).

			Non–Black/African American patients (n=149)				Black/African American patients (n=95)					*t* test (*df*)			*P* value
			Values, mean (SD)		95% CI		Values, mean (SD)		95% CI						
**Cancer health care experiences and perceptions^a^**															
	I feel confident in my decisions about treatment^b^.	8.57 (1.90)		8.26-8.88		8.32 (2.51)		7.81-8.83		0.836 (160.96)			.41		
	I have someone close to me I can talk to about my diagnosis and treatment options.	8.15 (2.97)		7.66-8.65		8.35 (2.72)		7.79-8.90		–0.508 (236)			.61		
	I have a lot of support from my family and friends.^b^	9.06 (1.94)		8.74-9.37		8.48 (2.69)		7.94-9.03		1.790 (157.03)			.08		
	I have a pastor or other religious leader that I trust and can talk to.	5.29 (3.96)		4.63-5.95		7.03 (3.88)		6.24-7.83		–3.336 (234)			.001		
	I have had someone close to me die of cancer.	8.12 (3.19)		7.59-8.64		7.55 (3.65)		6.80-8.30		1.260 (238)			.21		
	I have family members or close friends who have had cancer and been successfully treated.	7.58 (3.48)		7.01-8.15		6.59 (3.90)		5.79-7.39		2.038 (236)			.04		
	I trust the doctor treating me for my cancer.^b^	9.13 (1.70)		8.85-9.41		8.71 (2.38)		8.23-9.20		1.476 (153.83)			.14		
	It is important to get treated as soon as you are diagnosed to help prevent the cancer from coming back.^b^	9.26 (1.51)		9.01-9.51		9.05 (2.05)		8.63-9.47		0.849 (157.67)			.40		
	I researched information on my own about treatment options.	7.27 (3.11)		6.76-7.78		6.38 (3.55)		5.66-7.10		2.050 (237)			.04		
	I feel confident being able to research information on my own about treatment options.	6.65 (3.19)		6.12-7.17		6.78 (3.44)		6.07-7.48		–0.294 (237)			.77		
**Beliefs about health care providers and health^a^**															
	I go to the doctor for regular checkups.	9.11 (2.01)		8.78-9.44		9.04 (1.70)		8.70-9.39		0.271 (239)			.79		
	I get my cancer screenings whenever they are recommended.^b^	9.23 (1.66)		8.95-9.50		8.91 (2.02)		8.49-9.32		1.296 (173.79)			.20		
	Growing up we used a lot of home remedies.	3.56 (3.28)		3.02-4.10		6.03 (3.54)		5.30-6.77		–5.485 (235)			<.001		
	I believe using alternative therapies is important while being treated for cancer.	5.56 (3.38)		5.10-6.12		5.42 (3.86)		4.64-6.21		0.292 (236)			.77		
	I think that doctors mislead patients^b^.	1.37 (2.32)		0.99-1.75		2.50 (3.15)		1.85-3.15		–2.991 (156.97)			.003		
	I don’t trust medical researchers^b^.	1.23 (2.40)		0.83-1.62		2.69 (3.02)		2.07-3.31		–3.956 (167.01)			<.001		
	I believe racial/ethnic minorities are discriminated against in medical research studies^b^.	1.64 (2.66)		1.21-2.08		3.27 (3.46)		2.56-3.97		–3.876 (162.46)			<.001		
	I don’t trust drug (pharmaceutical) companies.	3.84 (3.27)		3.31-4.38		3.98 (3.25)		3.31-4.64		–0.316 (238)			.75		
**Patient activation in cancer care (DES-10^c^) ^a^**			8.00 (1.33)		7.78-8.22		7.85 (1.51)		7.54-8.15		0.841 (239)			.40	
**Patient self-advocacy (PSAS^d^) ^a^**			6.07 (1.49)		5.82-6.31		6.07 (1.69)		5.73-6.41		–0.009 (238)			.99	
**Health care self-efficacy (PEPPI^e^)**			4.45 (0.64)		4.34-4.55		4.44 (0.76)		4.28-4.59		0.109 (239)			.91	
**Decisional conflict^f^**															
	Certainty (range 0-100)	36.24 (25.76)		32.08-40.46		25.62 (22.17)		21.08-30.16		3.284 (237)			.001		
	Informed (range 0-100)	31.91 (20.75)		28.51-35.30		36.44 (24.95)		31.32-41.55		–1.523 (238)			.13		
	Values clarity (range 0-100)	37.84 (27.67)		33.32-42.37		41.31 (28.58)		35.46-47.17		–0.936 (238)			.35		
	Support (range 0-100)	21.52 (20.94)		18.09-24.94		22.61 (21.54)		18.19-27.02		–0.389 (238)			.70		
	Overall decisional conflict (range 0-100)	31.74 (19.80)		28.50-34.98		30.81 (19.66)		26.78-34.83		0.357 (238)			.72		
	Intentions to participate in clinical trial, if offered^a,b^	7.03 (2.60)		6.60-7.46		6.38 (3.16)		5.73-7.02		1.662 (174.01)			.10		

^a^Response options for perception items, patient activation, patient self-advocacy, and clinical trial intentions ranged from 0 to 10, where “0” indicated strong disagreement and “10” indicated strong agreement.

^b^Variances were not equal between groups, so an independent sample *t* test with unequal variances was used.

^c^DES-10: 10-item Decisional Engagement Scale.

^d^PSAS: Patient Self-Advocacy Scale.

^e^PEPPI: Perceived Efficacy in Patient-Physician Interactions; health care self-efficacy ranged from 1 to 5, where higher values reflected greater self-efficacy.

^f^Decisional conflict was a percentage ranging from 0 to 100.

### Sociodemographic and Clinical Characteristics

More than a third (95/244, 38.9%) of participants self-identified as Black/African American. Participants were aged a mean 60.89 (SD 10.24) years but did not vary by dichotomous race group. More than half (141/244, 57.8%) had at least some college or more, but educational attainment varied significantly between Black/African American and non–Black/African American participants (*P*<.001). Moreover, 63.1% (154/244) of the sample included female participants, but a greater percentage of the Black/African American patients were female (70/95, 73%) compared to the non–Black/African American patients (84/149, 56.4%; *P*=.006). Other significant differences between groups were observed for insurance type (ie, a greater percentage of Black/African American patients on Medicare or Medicaid), annual household income (ie, higher household income reported by non–Black/African American patients), and treatment status (ie, greater percentage of Black/African American patients still receiving treatment as opposed to follow-up care compared with non–Black/African American patients).

### General Clinical Trials Knowledge and Health Literacy

Compared to the Black/African American patients (mean 75.6, SD 12.7), the non–Black/African American patients (mean 80.7, SD 14.7) had significantly higher general clinical trial knowledge scores (t_242_=2.775; *P*=.006). Health literacy (greater values reflect lower health literacy) was also higher among non–Black/African American patients (mean 1.47, SD 0.72) than Black/African American patients (mean 2.06, SD 1.11; t_145.36_=−4.650; *P*<.001).

### Awareness of Cancer Clinical Trials

Non-Black patients (mean 7.61, SD 3.33) were significantly more likely to have heard about clinical trials before their cancer diagnosis compared with Black/African American patients (mean 5.19, SD 3.96; t_238_=5.075; *P*<.001). However, non–Black/African American patients (mean 5.93, SD 3.55) felt more strongly than Black/African American patients (mean 4.55, SD 3.63) that they did not have sufficient information to decide whether to participate in a cancer clinical trial (t_238_=2.920; *P*=.004). There were no differences between groups on all other awareness-related items, including information gathering, support for accessing and consuming cancer-related health information, and receiving sufficient information about cancer clinical trials from their health care providers.

### Benefits of Clinical Trial Participation

Black/African American patients consistently rated the benefits of cancer clinical trial participation lower than non–Black/African American patients. Specifically, Black/African American patients rated 10 out of 11 items about perceived benefits lower than non–Black/African American patients, all of which were statistically significant (*P* values were .02, .03, .03, .02, .001, .02, <.001, .04, .007, and .04). Benefits of cancer clinical trial participation rated lower included having better survival odds, improving quality of life, increasing access to high-quality treatment, having a greater sense of purpose, and helping to find treatments and cures for family members or the public. In fact, the only benefits-related item that did not yield significant differences between groups at α=.05 level was belief that clinical trial participation would improve their community’s trust in medical research (“Being part of a clinical trial will improve my community’s trust in medical research”).

### Concerns of Clinical Trial Participation

Concerns about cancer clinical trials that varied between racial groups were religious beliefs as barriers, fatalistic beliefs about cancer, and fears of receiving a placebo or sugar pill. Compared to non-Black patients, Black/African American patients with cancer were significantly more likely to believe that their religion or fatalistic beliefs (ie, “God has already decided what will happen so being part of a clinical trial would not help”) would keep them from participating in a clinical trial. However, non–Black/African American patients (mean 4.00, SD 3.66) were significantly more concerned than Black/African American patients (mean 2.72, SD 3.35) about potentially receiving a placebo and not real medicine (t_239_=2.750; *P*=.006).

### Cancer and Health Care Experiences

Religious leaders were more strongly endorsed as a form of social support for Black/African American patients than non-Black patients. For example, non-Black patients (mean 5.29, SD 3.96) were less likely than Black/African American patients (mean 7.03, SD 3.88) to say they have a pastor or other religious leader that they trusted and could talk to (t_234_=–3.336; *P*=.001). However, non-Black patients (mean 7.27, SD 3.11) were more likely to report independently researching treatment options than Black/African American patients with cancer (mean 6.38, SD 3.55; t_237_=2.050; *P*=.04). In addition, non-Black patients (mean 7.58, SD 3.48) more strongly endorsed agreement with having family or close friends who had been diagnosed with cancer and who were successfully treated than Black/African American patients (mean 6.59, SD 3.90; t_236_=2.038; *P*=.04).

### Beliefs About Health and Health Care Providers

Non-Black patients reported less frequent use of home remedies for medical care growing up than Black/African American patients (t_236_=–5.485; *P*<.001). In addition, 3 items of distrust of health care providers and medical mistrust were also endorsed more strongly by Black/African American patients (“I think that doctors mislead patients,” “I don’t trust medical researchers,” and “I believe racial/ethnic minorities are discriminated against in medical research studies”). However, ratings in both groups remained low and below a score of neutral (ie, “5”), reflecting overall low levels of medical mistrust in this sample.

### Patient Activation, Patient Self-Advocacy, and Health Care Self-Efficacy

There were no significant differences in average patient activation in cancer care, patient self-advocacy, or health care self-efficacy between Black/African American and non–Black/African American patients (all *P*>.05).

### Decisional Conflict

Of the 4 domains of decisional conflict, only certainty was significantly different between Black/African American and non–Black/African American patient groups. Black/African American patients with cancer (mean 25.62, SD 22.17) reported lower certainty in their clinical trial decision-making than non–Black/African American patients (mean 36.24, SD 25.76; t_237_=3.284; *P*=.001). The remaining 3 decisional conflict domains (informed, value clarity, and support) and summary decisional conflict score were nonsignificant between groups at the α=.05 level.

### Intentions to Participate in Clinical Trial, if Offered

Intentions to participate in a cancer clinical trial, if offered, did not differ significantly between Black/African American patients (mean 6.38, SD 3.16) and non–Black/African American patients (mean 7.03, SD 2.60) at the α=.05 level (t_174.01_=1.662; *P*=.10).

## Discussion

### Principal Findings

This analysis of baseline data from the mychoice randomized control study focused on patient perceptions regarding cancer clinical trials comparing Black/African American patients to non-Black patients. Some results are consistent with other research while also suggesting some unexpected findings that might shift the focus on how best to increase participation among Black/African American patients with cancer. Results indicate that addressing preparation for decision-making, community context, and the opportunity to reframe perceptions about interest in considering clinical trials are important constructs to target in efforts to reduce barriers to participation for Black/African American patients.

### Comparisons to Prior Work

Clinical trial decision-making is complex. As suggested by Wenzel et al [70], the Model of Cancer Clinical Trial Decision-Making provides a framework to explore these findings from the patient perspective including information gathering, intrapersonal and interpersonal factors that influence the decision-making process, all of which ultimately impact decisional outcomes.

Our findings suggest that there are differences at the start of the clinical trial decision-making process between Black/African American and non-Black patients. We found non–Black/African American patients had significantly higher levels of clinical trial knowledge, health literacy, and positive experiences with cancer outcomes, while Black/African American patients were less likely to hear about clinical trials before their diagnosis, creating inequities from the start. More challenging is combating the realities of later-stage disease at diagnosis and unequal oncology care in many communities of color where cancer outcomes are less positive [71,72]. These findings are consistent with the current literature and highlight the need for more community education and awareness about clinical trials using plain language and health communication approaches appropriate for all levels of health literacy [73]. As progress is made to address these inequities, it is important to emphasize these gains in our educational initiatives and share stories from survivors and clinical trial participants from these communities [74].

Our study findings are also consistent with other research highlighting that the potential benefits of participation are less likely to resonate with Black patients, including the notion that participation is a benefit to their community. One factor is a higher level of level of general medical mistrust found in the Black/African American community [75], which is associated with expectations of lower care quality and poorer treatment experiences [76].

Consistent with existing literature, Black/African American patients with cancer more frequently endorse fatalistic beliefs about the condition [77]. As noted in the model proposed by Wenzel et al [70], increased fatalism is an important factor in this decision-making process. Addressing these deep-rooted beliefs and experiences requires deeper, authentic discussions with community leaders, providers, and other stakeholders. Religious leaders, specifically, can be messengers to balance these beliefs because they can play an important role in individuals’ decision-making process [78]. To improve self-efficacy in cancer clinical trial decision-making and to improve clinical trial experiences overall, prior evidence-based recommendations have been made to establish long-term partnerships between not only the health care providers but also with other patients, patient advocates, researchers, clinical trial sponsors, and other community-based organizations (eg, faith-based groups and social services organizations) [55,79] as well as to form community advisory boards [80].

We found few differences in facilitators to clinical trial participation by race. Indeed, patients reported that they were confident in gathering support, trusted their physicians, and could get information from their physicians about clinical trials. Although general mistrust was more prevalent in Black/African American patients, their trust in their physician and their ability to get information about clinical trials was similar to non-Black patients. This was a much more nuanced view of medical mistrust and may vary significantly among Black/African American patients, depending on a range of sociodemographic factors and life experiences. In addition, it is important to note that general mistrust might be mitigated by the providers providing direct care, which could include providers from a variety of specialties and primary care. Therefore, initiatives and interventions to educate a broad range of providers about clinical trials and emphasize their role in this decision-making process are essential to increasing participation.

An unexpected finding was that non-Black patients reported higher levels of concerns about receiving a placebo and felt they did not have sufficient information to decide about participation. This may be related to their higher levels of clinical trial knowledge that might initially raise more questions and concerns, recognizing the complexity of the process. As more comprehensive education is conducted in Black/African American communities, we might expect that these will be issues that need to be specifically addressed.

Perhaps most importantly, there were no differences between Black/African American and non-Black patients in their intention to participate if offered a clinical trial. This was true despite having found important differences in perceived barriers to participation by race. However, provider and system barriers may impact the ability of patients to turn intention into decision-making and participation. If a trial were available and yet not offered, there is an unwarranted bias that they would not be interested. If a trial is not available, then there is no decision to make. This expands the Wenzel model beyond the patient [70], focusing on the multilevel influences on this decision-making process. Future research could include both the mychoice patient tool and provider training and interventions to increase cultural competency and change the knowledge and attitudes of providers and study staff, as well as providing culturally tailored education initiatives to increase education and awareness of clinical trials among racial and ethnic minoritized populations [81-83]. Our own work developing the mychoice web-based tool to assist diverse patients in the decision-making process serves as an example [58].

### Future Directions

We recognize that patients’ knowledge, attitudes, and interest in clinical trial participation are only one facet of this complex process. Availability of clinical trials in local settings, systematic barriers to care, language and cultural barriers, provider attitudes, and trial eligibility requirements all must be addressed as well. To date, many programs and interventions have been implemented at multiple levels or at the organization or systems levels to address systemic factors that drive the continued underrepresentation of people from racial and ethnic minoritized groups in research. For example, 1 system-level approach is the creation of the US Cancer Centers of Excellence and an inventory of successful strategies for increased inclusion of people from racial and ethnic minoritized groups in clinical trials [84,85]. Specifically, leaders from 8 US cancer centers met to determine best practices for increasing enrollment and retention of clinical trial participants from racial and ethnic minoritized groups. Topics discussed included hiring practices; cultural changes in research organizations; and education or training on equity, diversity, and inclusion among people who study and work in cancer clinical trials [55,84,85]. These changes are important because patient-provider identity concordance can motivate greater interpersonal trust, cancer care engagement, and care quality [86-88], yet Black oncologists remain significantly underrepresented within the health care workforce, with Black oncologists making up only 3% of all oncologists in the United States as of 2021 [87].

Finally, studies should also publish data more frequently on the racial and ethnic composition of their study participants in their published clinical trial reports and in registry results [55]. While applicable to public health and medical fields beyond oncology, increased transparency about the demographic composition of clinical trials will assist with monitoring of diversity, equity and inclusion progress and support future meta-analytic research. For example, among the 197 precision oncology clinical trials in the United States from 2004 to 2017 reported on ClinicalTrials.gov, fewer than half (n=97, 49.2%) provided race or ethnicity data [4]. Similarly, recent systematic reviews found that only 57% of the 155 head and neck cancer clinical trials between 2010 and 2020 [89] and only 4.4% of the 544 bladder cancer clinical trials published between 1970 and 2020 had race or ethnicity demographic data [90].

### Limitations

This study has several limitations. First, this was a cross-sectional analysis that limits inferences to causality. Second, generalizability is limited to people already receiving care for cancer. This is noteworthy because cancer disparities exist before this point (ie, detection, treatment provision, etc), meaning that there may be different beliefs and attitudes associated with patients who have not engaged with cancer treatment services. This may also limit generalization to some specific patient populations, such as recent immigrants, without adequate health insurance and health care access. Moreover, this was a baseline sample of patients diagnosed with cancer recruited from cancer treatment centers for an RCT. Thus, this sample of participants likely already has higher acceptance of clinical trials because they had already consented to be in a behavioral trial. In addition, results also suggest that these participants may have higher acceptance of Western medicine and health care providers because they are already receiving care at a cancer treatment center. This sample reported low levels of health care provider medical mistrust and few reports of negative health care experiences across both the Black/African American and non-Black groups, which is likely not representative of the US adult cancer population, especially Black adults [46,91,92].

While social desirability bias can contribute to underreporting of negative health care experiences and other negative health care attitudes and beliefs, the web-based, self-administered survey format may have mitigated the extent to which social desirability bias could have impacted the validity of participant responses. Another potential limitation is that these analyses did not control for multiple comparisons made on the same data set. While some researchers suggest using the Bonferroni adjustment to control for the possibility of finding false positives when making multiple comparisons, there is criticism of its unilateral use in multiple comparison studies [93]. That said, there remains some potential for inflated type 1 error (ie, false positives) given the number of hypotheses tested. Finally, there are also additional barriers to cancer clinical trial participation that are not accounted for in the present analysis. For example, older age, insurance type (ie, Medicaid and uninsured vs private insurance), greater medical comorbidities, and greater distance to treatment are associated with lower rates of clinical trial participation [94] and high-quality, guideline-concordant cancer care [95]. Thus, covariate-adjusted analysis methods should be considered for subsequent work.

### Conclusions

The findings from the baseline survey of the mychoice randomized trial highlight that although clinical trial participation among diverse populations remains low, there were no significant differences in interest in clinical trials, and trust in individual providers was high in both Black/African American and non-Black patients with cancer. However, persistent beliefs about barriers to and benefits of participation in clinical trials exist. Our findings suggest that we need more outreach, discussion, and introduction of clinical trials to diverse oncology patients who may be more interested than presumed. This does not preclude the considerable work that needs to be done to address access to clinical trials and addressing the systemic barriers to participation. Importantly, the findings from this study suggest that current interventions have not significantly moved the needle in broadening the appeal of clinical trials in Black/African American patients with cancer, and further work in effectively increasing participation rates is still needed.

## Abbreviations

RCT: randomized controlled trial
REDCap: Research Electronic Data Capture

## Data Availability

The data sets generated during and analyzed during this study are not publicly available because they contain information that could potentially identify patients but are available from the corresponding author on reasonable request.
